# Epigenetic Therapy in Human Choriocarcinoma

**DOI:** 10.3390/cancers2031683

**Published:** 2010-09-10

**Authors:** Noriyuki Takai, Hisashi Narahara

**Affiliations:** 1 Department of Obstetrics and Gynecology, Oita University Faculty of Medicine, Oita, Japan; 2 Department of Obstetrics and Gynecology, Oita University Faculty of Medicine, 1-1 Idaigaoka, Hasama-machi, Yufu-shi, Oita 879-5593, Japan; E-Mail: naraharh@oita-u.ac.jp

**Keywords:** choriocarcinoma, epigenetics, histone deacetylase inhibitors

## Abstract

Because epigenetic alterations are believed to be involved in the repression of tumor suppressor genes and promotion of tumorigenesis in choriocarcinomas, novel compounds endowed with a histone deacetylase (HDAC) inhibitory activity are an attractive therapeutic approach. HDAC inhibitors (HDACIs) were able to mediate inhibition of cell growth, cell cycle arrest, apoptosis, and the expression of genes related to the malignant phenotype in choriocarcinoma cell lines. In this review, we discuss the biologic and therapeutic effects of HDACIs in treating choriocarcinoma, with a special focus on preclinical studies.

## 1. Introduction

Gestational choriocarcinoma, a group of rare placenta disorders, have varying potential for invasion, either local, or remote in the form of metastases. Women with gestational choriocarcinoma who fail to respond to well-established first-line chemotherapy have an extremely poor prognosis in spite of multiagent chemotherapy [1,2,3]. Definitive second-line and third-line chemotherapy regimens remain to be identified, and newer cytotoxic agents are of interest in this regard.

One of the most important mechanisms in chromatin remodeling is the post-translational modification of the *N*-terminal tails of histones by acetylation, which contributes to a ‘histone code’ determining the activity of target genes [4]. Transcriptionally silent chromatin is composed of nucleosomes in which the histones have low levels of acetylation on the lysine residues of their amino-terminal tails. Acetylation of histone proteins neutralizes the positive charge on lysine residues and disrupts the nucleosome structure, allowing unfolding of the associated DNA with subsequent access by transcription factors, resulting in changes in gene expression. Acetylation of core nucleosomal histones is regulated by the opposing activities of histone acetyltransferases (HATs) and histone deacetylases (HDACs). HDACs catalyze the removal of acetyl groups on the amino-terminal lysine residues of core nucleosomal histones, and this activity is generally associated with transcriptional repression. HDACs remove the acetyl groups which then induce a positive charge on the histones, and this suppresses gene transcription, including tumor suppressor genes silenced in cancer. Moreover, acetylation of histones facilitates destabilization of DNA-nucleosome interactions and renders DNA more accessible to transcription factors [5]. Aberrant recruitment of HDAC activity has been associated with the development of certain human cancers [6]. HDAC inhibitors (HDACIs) can inhibit cancer cell growth *in vitro* and *in vivo*, revert oncogene-transformed cell morphology, induce apoptosis, and enhance cell differentiation.

The classes of HDACIs that have been identified are: (a) organic hydroxamic acids [e.g., Trichostatin A (TSA) and suberoylanilide bishydroxamine (SAHA)]; (b) short-chain fatty acids [e.g., butyrates and valproic acid (VPA)]; (c) benzamides [e.g., MS-275]; (d) cyclic tetrapeptides [e.g., trapoxin]; and(e) sulfonamide anilides [7] (see Table 1).

**Table 1:** Overview of frequently used histone deacetylase inhibitors that are available for clinical and research purposes.

Substance groups	Derivatives	Isotype
Hydroxamates	Trichostatin A (TSA)	I, II
	Suberoylanilide hydroxamic acid (SAHA, vorinostat)	I, II, IV
	LBH589 (panobinostat)	I, II, IV
	PCI24781 (CRA-024781)	I, IIb
	LAQ824	I, II
	PXD101 (belinostat)	I, II, IV
	ITF2357	I, II
	SB939	Unknown
	JNJ-16241199 (R306465)	I
	m-carboxycinnamic acid bishydroxamide (CBHA)	
	Scriptaid	
	Oxamflatin	
	Pyroxamide	
	Cyclic hydroxamic acid containing peptides (CHAPs)	
Short chain fatty acids	Butyrate	I, IIa
	Valproate	I, Iia
	AN-9	
	OSU-HDAC42	
Benzamides	MS-275 (entinostat)	1, 2, 3, 9
	MGCD0103	1, 2, 3, 11
	Pimelic diphenylamide	1, 2, 3
	M344	
Cyclic tetrapeptides	Apicidine	I, II
	Trapoxins	
	HC-toxin	
	Chlamydocin	
	Depsipeptide (FR901228 or FK228) (romidepsin)	1, 2, 4, 6
Sulfonamide anilides	N-2-aminophenyl-3-[4-(4-methylbenzenesulfonylamino)-phenyl]-2-propenamide	
Others	Depudecin	
	NDH-51	
	KD5150	Pan-HDACI

Class I: HDAC1, -2, -3 and -8; class IIa: HDAC4, -5, -7, and -9; class IIb: HDAC 6, and -10; class III: SIRT1-7; class IV: HDAC11

At least 80 clinical trials are underway, testing more than 11 different HDAC inhibitory agents including both hematological and solid malignancies [8,9,10]. However, there is no clinical trial for treating choriocarcinoma. In this review, we discuss the biologic and therapeutic effects of HDACIs in treating choriocarcinoma, with a special focus on preclinical studies.

## 2. Mechanism of Action

Histone deacetylases (HDACs) comprise a family of 18 genes that are subdivided into four classes [11]. Classes I, II, and IV are referred to as ‘‘classical” HDACs and are generally simultaneously targeted by most HDACIs (Table 1). HDACIs were initially discovered on the basis of their ability to reverse the malignant phenotype of transformed cells in culture. It has been shown that HDACIs carry the potential to activate differentiation programs on one hand, while on the other hand they were also shown to inhibit cell proliferation by cell cycle arrest in the G1 and/or G2 phases of the cell cycle and to induce apoptosis in cultured transformed cells. p21^WAF1^ and p27^KIP1^ are cyclin-dependent kinase inhibitors (CDKIs) that bind to cyclin-dependent kinase complexes and decrease kinase activity, and may act as key regulators of the G0/G1 accumulation (reviewed in [12]). The p21^WAF1^ expression in particular is induced by HDACIs in various cell lines. Additionally, this event is associated with both an increase in histone acetylation in the promoter region of the p21^WAF1^ gene and a selective loss of a specific HDAC enzyme, HDAC1, in the same region [13]. Therefore, the upregulation of p21^WAF1^ is a direct consequence of HDACIs on p21^WAF1^ transcription. In the future, testing should be conducted using p21^WAF1^-negative cell lines to see if p21^WAF1^ is absolutely required for HDACI-induced growth arrest. Takai *et al.* examined the effect of HDACIs on the expression of p21^WAF1^ and p27^KIP1^ in choriocarcinoma cells by Western blot analysis. HDACIs markedly upregulated the level of p21^WAF1^ and p27^KIP1^ proteins, which were expressed at negligible levels in the untreated cell lines. Conversely, HDACIs decreased the levels of cyclin A. HDACIs decreased the bcl-2 levels. E-cadherin binds to β-catenin and can act as a tumor suppressor gene; its promoter has CpG islands, which are frequently methylated in selected cancers. HDACIs markedly increased the expression level of E-cadherin in choriocarcinoma cells and exhibited antiproliferative activity in these cells [14]. HDACIs have also been shown to generate reactive oxygen species (ROS) in solid tumor and leukemia cells [15,16,17], which may contribute to the mechanism. HDACIs have been shown to inhibit angiogenesis. HDACIs repress the expression of proangiogenic factors such as HIF1α, VEGF, VEGF receptor, endothelial nitric oxide synthase, IL-2 and IL-8 and the induction of antiangiogenic factors, such as p53 and von Hippel-Lindau (reviewed in Ref. [18]). HDACIs should not be considered to act solely as enzyme inhibitors of HDACs. A large variety of non–histone transcription factors and transcriptional co-regulators are known to be modified by acetylation. HDACIs can alter the degree of acetylation of non–histone effector molecules and thereby increase or repress the transcription of genes by this mechanism. Examples include: ACTR, cMyb, E2F1, EKLF, FEN 1, GATA, HNF-4, HSP90, Ku70, NF-κB, PCNA, p53, RB, Runx, SF1 Sp3, STAT, TFIIE, TCF, YY1, *etc.* (reviewed in Ref. [18]).

## 3. Preclinical Epigenetic Therapy in Choriocarcinoma

A variety of structurally distinct classes of compounds that inhibit deacetylation of both histone and non-histone proteins have gradually been identified (Table 1). Despite the shared capacity of each class of HDACIs to promote histone acetylation, individual HDACIs exert different actions on signal transduction and the induction of differentiation and/or apoptosis. Takai *et al.* investigated the effect of different classes of HDACIs in the BeWo choriocarcinoma cell line [14]. BeWo cells showed significant sensitivity to VPA with 1.2 × 10^−3^ M, CBHA with 2.0 × 10^−6^ M, M344 with 7.8 × 10^−6^ M, MS-275 with 6.3 × 10^−6^ M, and Scriptaid with 2.2 × 10^−6^ M, respectively, which caused a 50% inhibition (ED50) of their growth. Cell cycle analysis indicated that their exposure to HDACIs decreased the proportion of cells in the S-phase and increased the proportion in the G0/G1 phases of the cell cycle. Induction of apoptosis was confirmed by annexin V staining of externalized phosphatidylserine and loss of the transmembrane potential of mitochondria [14].

Senescence is an irreversible proliferative arrest which is induced by a number of stressors including oxidative DNA damage as well as oncogenes. Gregorie *et al.* elucidated that TSA treatment induces the senescence-associated β-galactosidase (SA-β-gal) marker in JAR choriocarcinoma cells [19]. The percentages of SA-β-gal-positive-JAR cells were 41% when cells were treated with 25 nM TSA for 24 h, 67% in cells 72 h after 5Gy of irradiation (positive control) and 9% in untreated negative control cells [19].

A DNA methyltransferase inhibitor, 5’-aza-2’-deoxycytidine (AZA), prevented BeWo cell migration in wound healing and transwell migration assays. AZA consequently inhibited BeWo cell invasion through reconstituted basement membrane. Treatment of BeWo cells with AZA resulted in conversion of cell morphology to a nonmigratory phenotype [20].

Although we would like to specifically discuss the effects of HDAC inhibitors or epigenetic agents on treating choriocarcinoma, there are no more extensive preclinical studies related to choriocarcinoma. Some potential strategies combining HDAC inhibitors or epigenetic agents with other systemic therapies for choriocarcinoma should be interesting.

## 4. Conclusions and Future Directions

In this review, we summarize recent preclinical studies on the use of HDACIs, especially in human choriocarcinoma cells. Further work is needed to improve our understanding of why transformed cells are more susceptible to the effect of HDACIs than normal cells. Also, combinations of HDACIs with differentiation-inducing agents, with cytotoxic agents, and even with gene therapy may represent novel therapeutic strategies and new hope for the treatment of choriocarcinoma.

## 5. Conflict of Interest

We declare that we have no conflict of interest.
