# Expression of Concern: Takai, N. and Narahara, H. Epigenetic Therapy in Human Choriocarcinoma. *Cancers* 2010, *2*, 1683–1688

**DOI:** 10.3390/cancers8040044

**Published:** 2016-04-07

**Authors:** Cancers Editorial Office

**Affiliations:** MDPI AG, Klybeckstrasse 64, CH-4057 Basel, Switzerland; cancers@mdpi.com; Tel.: +41-61-683-77-34

We wish to make readers aware that text in [1] has been taken from other publications by the same author. In particular, much of Section 1, the first part of Section 2 and the first sentence of Section 3 are copied verbatim from [2]. Table 1 and the second part of Section 2 are taken from [3]. The main novel contribution of [1] lies in Section 3. We regret that these issues were not picked up before publication and wish to apologize to readers of *Cancers*.
